# Enhancing Tropane Alkaloid Production Based on the Functional Identification of Tropine-Forming Reductase in *Scopolia lurida*, a Tibetan Medicinal Plant

**DOI:** 10.3389/fpls.2017.01745

**Published:** 2017-10-16

**Authors:** Kaihui Zhao, Junlan Zeng, Tengfei Zhao, Haoxing Zhang, Fei Qiu, Chunxian Yang, Lingjiang Zeng, Xiaoqiang Liu, Min Chen, Xiaozhong Lan, Zhihua Liao

**Affiliations:** ^1^Key Laboratory of Eco-Environments in Three Gorges Reservoir Region (Ministry of Education), Chongqing Key Laboratory of Plant Ecology and Resources Research in Three Gorges Reservoir Region, SWU-TAAHC Medicinal Plant Joint R&D Centre, School of Life Sciences, Southwest University, Chongqing, China; ^2^TAAHC-SWU Medicinal Plant Joint R&D Centre, Tibetan Collaborative Innovation Centre of Agricultural and Animal Husbandry Resources, Tibet Agricultural and Animal Husbandry College, Nyingchi, China; ^3^College of Life Sciences and Oceanography, Shenzhen University, Shenzhen, China; ^4^SWU-TAAHC Medicinal Plant Joint R&D Centre, College of Pharmaceutical Sciences, Southwest University, Chongqing, China; ^5^State Key Laboratory Breeding Base of Dao-di Herbs, National Resource Centre for Chinese Materia Medica, China Academy of Chinese Medical Sciences, Beijing, China

**Keywords:** enzymatic assay, metabolic engineering, *Scopolia lurida*, tropane alkaloids, tropine-forming reductase

## Abstract

*Scopolia lurida*, a native herbal plant species in Tibet, is one of the most effective producers of tropane alkaloids. However, the tropane alkaloid biosynthesis in this plant species of interest has yet to be studied at the molecular, biochemical, and biotechnological level. Here, we report on the isolation and characterization of a putative short chain dehydrogenase (SDR) gene. Sequence analysis showed that SlTRI belonged to the SDR family. Phylogenetic analysis revealed that SlTRI was clustered with the tropine-forming reductases. *SlTRI* and the other TA-biosynthesis genes, including *putrescine N-methyltransferase* (*SlPMT*) and *hyoscyamine 6*β*-hydroxylase* (*SlH6H*), were preferably or exclusively expressed in the *S*. *lurida* roots. The tissue profile of *SlTRI* suggested that this gene might be involved in tropane alkaloid biosynthesis. By using GC-MS, SlTRI was shown to catalyze the tropinone reduction to yield tropine, the key intermediate of tropane alkaloids. With the purified recombinant SlTRI from *Escherichia* *coli*, an enzymatic assay was carried out; its result indicated that SlTRI was a tropine-forming reductase. Finally, the role of SlTRI in promoting the tropane alkaloid biosynthesis was confirmed through metabolic engineering in *S*. *lurida*. Specifically, hairy root cultures of *S*. *lurida* were established to investigate the effects of SlTRI overexpression on tropane alkaloid accumulation. In the *SlTRI*-overexpressing root cultures, the hyoscyamine contents were 1.7- to 2.9-fold higher than those in control while their corresponding scopolamine contents were likewise elevated. In summary, this functional identification of *SlTRI* has provided for a better understanding of tropane alkaloid biosynthesis. It also provides a candidate gene for enhancing tropane alkaloid biosynthesis in *S*. *lurida* via metabolic engineering.

## Introduction

*Scopolia lurida*, also known as Himalayan Scopolia (Mills and Jackson, 1972) is a perennial herb of the family Solanaceae and indigenous to the Himalayas of the Qinghai-Tibet Plateau (Qin et al., 2014). Some Solanaceae species including *Hyoscyamus niger* (Yun et al., 1992), Datura species (Jakabová et al., 2012), *Atropa belladonna* (Wang et al., 2011), and *S. lurida* (Wang et al., 2010) are widely used as anticholinergic agents, especially the pharmaceutical tropane alkaloids (TAs), such as hyoscyamine and scopolamine that are produced exclusively by the medicinal plant family. Among these TA-producing plant species, *S*. *lurida* is one of the most effective producers of TAs (Jovanović et al., 1991) because of its high biomass and content of hyoscyamine which was reported as up to 1.5% dry weight in the aerial parts (Mills and Jackson, 1972). The scopolamine content in *S. lurida* was also higher than that in *A. belladonna* (Zeng et al., 2016). Therefore, *S*. *lurida* is not only a valuable plant source for commercially producing tropane alkaloids but also an interesting plant for studying TA biosynthesis. Unfortunately, biosynthesis and regulation of TAs in *S*. *lurida* is largely unknown at the molecular, biochemical and biotechnological level.

**FIGURE 1:**
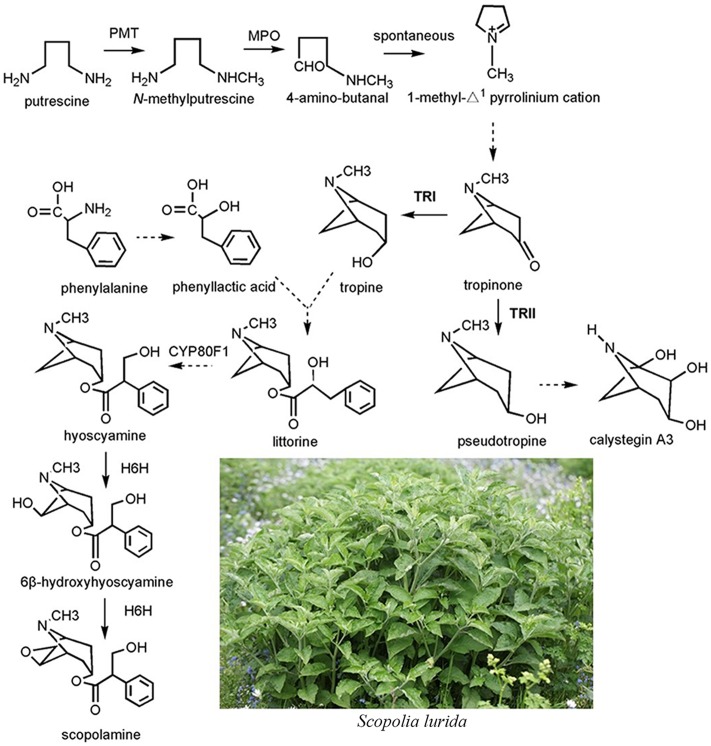
The biosynthetic pathway of tropane alkaloids in the plant species of Solanaceae.

Although the complete biosynthetic pathway of TAs is still unclear, several enzymes have been identified (**Figure 1**). Putrescine *N*-methyltransferase (PMT), the first committed enzyme of TA biosynthesis, catalyzes putrescine to form *N*-methylputrescine (Zhang et al., 2007). Overexpression of the tobacco PMT gene promotes TA accumulation in *Datura metel* root cultures (Moyano et al., 2003). After a series of enzymatic steps, the tropinone production in plants consists of a multistep enzymatic reaction. Two sequence-similar tropinone reductases (TRs) constitute a branch point in the TA metabolism pathway; both catalyze the stereospecific reduction of the 3-carbonyl group of tropinone to hydroxyl groups with different stereospecific configurations (Nakajima and Hashimoto, 1999). One such TR is the tropine-forming reductase (TRI) that converts tropinone to tropine incorporated into TAs, such as hyoscyamine and scopolamine; the other is pseudotropine-forming reductase (TRII), which reduces tropinone to pseudotropine that participates in the biosynthesis of nortropane alkaloids, including the calystegines (Nakajima and Hashimoto, 1999). To date, TRs and their coding genes have been identified from two well-known TA-producing plant species, *D*. *stramonium* and *H*. *niger*, at the molecular and biochemical level (Dräger et al., 1988; Nakajima et al., 1993a,b) TRI clearly plays an important role in TA biosynthesis. In hairy root cultures of *A*. *belladonna*, overexpression of TRI from *D*. *stramonium* was accompanied by a three and fivefold increase, respectively, in hyoscyamine and scopolamine contents relative to the controls of some transgenic root cultures (Richter et al., 2005). Hyoscyamine 6β-hydroxylase converts hyoscyamine to scopolamine, the last rate-limiting enzyme in the TA biosynthetic pathway. Without exception, overexpression of H6H leads to the enhancement of scopolamine production (Zhang et al., 2005).

To date, however, no TA biosynthesis genes from Himalayan Scopolia have been reported. To reveal the biosynthesis and regulation of TA biosynthesis in *S*. *lurida*, we cloned a putative TRI gene from *S*. *lurida*, characterized it at molecular level, and functionally identified it at the biochemical level. Finally, we used this gene to genetically modify the TA biosynthetic pathway in the root cultures of *S*. *lurida*.

## Materials and Methods

### Plant Materials

The seeds of *S*. *lurida* were taken from the 4000-m-high northern slope of the Shergyla Mountain of Nyingchi (Tibet, China), in September 2013. The seedlings germinated from these seeds grew in pots containing a substrate mixture (pearlite: vermiculite: Pindstrup Moss Peat = 1:1:2) in a greenhouse (16-h light). When the seedlings had grown 8–10 cm in height, their leaves, stems, and roots were harvested and immediately immersed in liquid nitrogen for later use. These seedlings were bacteria-free, which were established following the method we recently reported (Qin et al., 2014).

### Gene Cloning and Expression Analysis

Total RNAs were, respectively, isolated from the frozen leaves, stems, roots, and hairy roots by using the Plant RNA kit (TianGen, Beijing, China). RNA from the roots was used as the initial material for gene cloning. First-strand cDNAs were synthesized with a PrimeScript RT Kit (TaKaRa, Dalian, China). A pair of degenerate primers, *fdSlTRI* and *rdSlTRI*, were used for amplifying the *SlTRI* fragment. Based on the ensuing amplified fragments, the gene-specific primers were then used to amplify the *SlTRI*. For SlTRI, both *SlTRI3-1* and the M 13 Primer M4 were used to amplify the 3′-end; then, the two primers *SlTRI3-2* and M 13 Primer M4 were used to nest the amplified 3′-end. The corresponding fragments were assembled to generate the putative cDNA sequences. Finally, the physical sequences of SlTRI were amplified, by using two gene-specific primers, *fSlTRI* and *rSlTRI*. Three clones for *SlTRI* were sequenced to confirm the gene sequences. The amino acid sequences were deduced with help of ORFinder and their corresponding theoretical molecular weights were calculated by Vector NTI software. BLASTP was used to analyze the sequence similarity^1^; ClustalX (Larkin et al., 2007) and MEGA (Tamura et al., 2011) were used for the phylogenetic analysis. The relative gene expression levels were detected by real-time quantitative PCR (qPCR) in the leaves, stems, roots, transgenic, and non-transgenic hairy root cultures of *S*. *lurida*. The *phosphoglycerate kinase* gene (PGK) served as the internal reference. For the qPCR analysis, the kits purchased from Bio-Rad were used; the qPCR system was Bio-Rad IQ5 (Bio-Rad, Hercules, CA, United States). We used our previously reported method for the gene expression analysis (Qin et al., 2014). The primers for the gene expression analysis are listed in **Table 1**. The data were collected from at least three biological repeats.

**Table 1:** The primers used in this study.

Primer name	Primer sequences (5′→3′)
*fdSlTRI*	CATATCTCAAAATADTWRCAAG
*rdSlTRI*	TYAAAAYCCACCATTAGCWG
*SlTRI3-1*	GAATGGGCCAAGGAGAAC
*SlTRI3-2*	TTCTTTGCTTCCCTGCTGC
*SlPMT* -qRT-F	ACCAGCCATGTCAAATCCAAGG
*SlPMT* -qRT-R	TCCCCATACACAACCAAACGTG
*SlTRI* -qRT-F	TTCTGTTTTCTGTTTGGGCTTTG
*SlTRI* -qRT-R	GCTGACACAAGAATCATCTAGGC
*SlH6H* -qRT-F	F:GACTACATCTGTGAAGGACTTG
*SlH6H* -qRT-R	CAGCAATCCAGTTGTCATCC
*SlPGK*-qRT-F	GCTGCTGGAACGGAGGCTATTG
*SlPGK*-qRT-R	TGTGGCTCATCTTCTCTGCAAGTC
*SlTRI* -pET-F	CGGATCCATGGGAGAATCAAAAGTTTAC
*SlTRI* -pET-R	CGAGCTCTCAAAACCCACCATTAGCTGT
*SlTRI* -ovx-F	CGGATCCATGGGAGAATCAAAAGTTTAC
*SlTRI* -ovx-R	CGAGCTCTCAAAACCCACCATTAGCTGT
*NPTII*-F	CCAACGCTATGTCCTGATAG
*NPTII*-R	CTGAATGAACTCCAGGACGAG
*rolB*-F	GCTCTTGCAGTGCTAGATTT
*rolB*-R	GAAGGTGCAAGCTACCTCTC
*rolC-*F	TAACATGGCTGAAGACGACC
*rolC-*R	AAACTTGCACTCGCCATGCC

### Purification and Enzymatic Assay of Recombinant Protein

To obtain the recombinant protein, the coding sequence of SlTRI was inserted into the protein expression vector, pET-28a, within the restriction enzymes of *Bam*HI and *Sac*I. The primers used for constructing the protein recombination vector are listed in **Table 1**. The *Escherichia* *coli* strain Rosetta was used as the host cells for expressing the recombinant protein. The methods of producing and purifying the recombinant proteins followed those of Qiang et al. (2016). The tropinone feeding assay was used to identify the function of SlTRI. The total reaction volume of 10 ml was composed of 100 mM of tropinone, 200 μM of NADPH, 100 mM buffer of potassium phosphate, and 5 μg of the recombinant protein. After incubating for 2 h at 30°C, the reaction products were extracted as described by Kushwaha et al. (2013) and they were analyzed by GC-MS (QP2010 Ultra, Shimadzu, Kyoto, Japan) according to published methods (Kushwaha et al., 2013). Briefly, the GC-MS was equipped with an Rtx-5 (5% diphenyl) dimethylpolysiloxane capillary column (30 m, 0.25 mm ID, 0.25 μm df). Helium was used as the carrier gas, with a constant flow rate set at 1 ml min^-1^. The injection volume was 1 μl with a split ratio of 20:1. The inlet temperature was set at 250°C. The temperature of the flame ionization detector was set at 300°C. The GC program went as follows: 65°C as the initial temperature, up to 150°C (10°C/min), isotherm for 2 min, then up to 240°C (10°C/min), and finally, at 240°C for 5 min.

### Establishment of the Hairy Root Cultures and PCR Detection

The coding sequence of *SlTRI* was inserted into pBI121 within the *Bam*HI and *Sac*I. Then, the constructs harboring *SlTRI* were introduced into the *Agrobacterium* C58C1 (pRiA4) that was used for genetic transformation of *S*. *lurida*. The primers used for constructing the plant expression vectors are listed in **Table 1**. Hairy roots were induced from the bacteria-free leaves of *S*. *lurida*, as we did previously (Qin et al., 2014). The root lines without bacterial contamination were cultivated in a 100-ml liquid MS medium in 250 ml flasks (110 rpm) on an orbital shaker at 25°C in the dark. The *SlTRI*-overexpressing root cultures and the control root cultures were harvested after 40 days of cultivation. The control root cultures came from three independent hairy root lines not transformed by *SlTRI*. There were three biological replicates in these experiments. The kanamycin-resistant gene, as well as two rooting genes, *rolB* (*root loci B*) and *rolC* (*root loci C*), were detected as done in our other work (Qin et al., 2014; Xia et al., 2016). To confirm the authentic transgenic root cultures, the specific fragment of the transgene (*SlTRI*) fused with the 35S promoter was amplified, and the primers for this are listed in **Table 1**.

### Alkaloid Analysis by High Performance Liquid Chromatography

The experimental plant materials, including the leaves, stems, roots, transgenic root cultures, and control root cultures, were thoroughly dried at 40°C and then ground into a fine powder for use in the extraction of alkaloids (Xia et al., 2016) and their HPLC analysis (Wang et al., 2011). This alkaloid analysis was performed on the LC-20A system of HPLC with a photo-diode array detector (Shimadzu, Kyoto, Japan), while a CTP-ODS column (Phenomenex, Torrance, CA, United States) was used at 40°C with a 226-nm detecting wavelength. A sample of 20 μl was injected each time. The authentic samples included the hyoscyamine and scopolamine, as purchased from Sigma–Aldrich (St. Louise, MO, CA, United States).

## Results

### Gene Cloning and the Sequence Analysis

**FIGURE 2:**
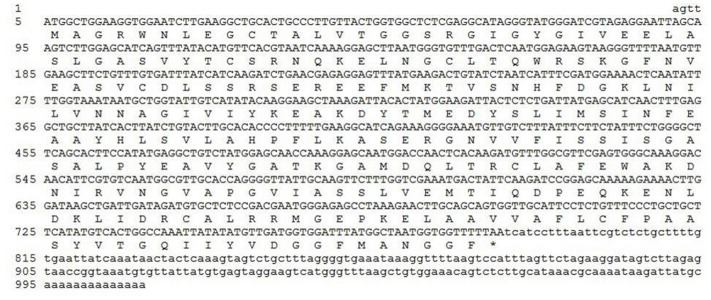
The full-length cDNA of SlTRI and its deduced amino acid sequence. The coding sequence and peptide sequence are shown in capital letters. ^∗^Represents the stop codon.

**FIGURE 3:**
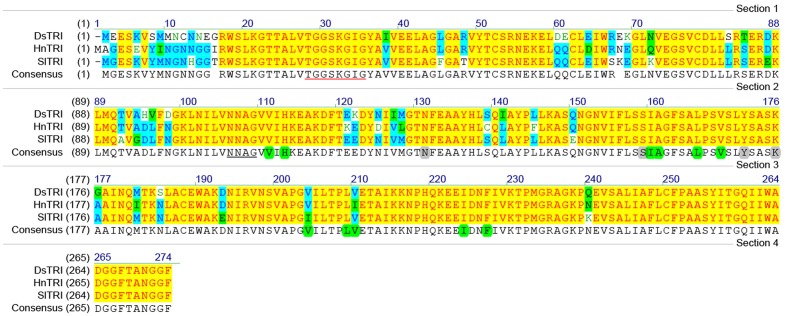
The amino acid sequence alignments for the plant tropinone reductases (TRs) that were functionally identified in this study. The NADPH-binding site (TGXXXGXG) is underlined in red. The SDR catalytic tetrad motif, characterized by N-S-Y-K, is highlighted in gray. The active amino acids of TRs are highlighted in green. DsTRI: *Datura stramonium* TR I; HnTRI: *Hyoscyamus niger* TR I; SlTRI: *Scopolia lurida* TR I.

**FIGURE 4:**
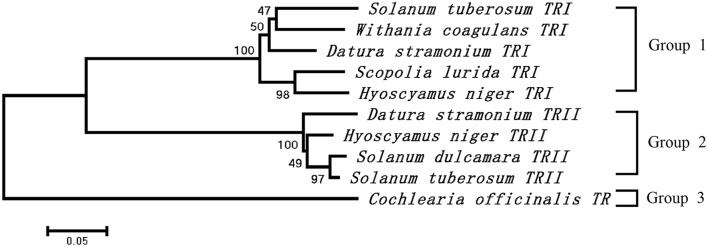
Phylogenetic analysis of the plant TRs that were functionally identified in this study. The numbers on the phylogenetic tree were calculated based on 1000 repeats.

The cDNA of SlTRI was 1148 bp in length, encoding a 29.4 kDa polypeptide that contained 273 amino acids (**Figure 2**). The BLASTP analysis showed that SlTRI belonged to the family of SDRs, the NADPH-dependent short chain dehydrogenases/reductases (Dräger, 2006). At the sequence level, SlTRI was highly similar to the tropine-forming reductases from *D*. *stramonium* (Portsteffen et al., 1994) and *H*. *niger* (Dräger et al., 1988). The SDR-specific motifs were found in SlTRI (**Figure 3**). Serving as the NADPH-binding site, the conserved TGXXXGXG motif (Oppermann et al., 2003) was present at the 27–34 position in SlTRI. The signature motif of SDRs was found at the 105–108 position in SlTRI. SlTRI contained the catalytic tetrad motif characterized by N-S-Y-K, in which the tyrosine (Y) residue is considered essential for the catalytic activity of TRs (Nakajima et al., 1999; Qiang et al., 2016). The bioinformatics analysis suggested that SlTRI might be one of the TRs, and the follow-up phylogenetic analysis gave clear clues to SlTRI’s classification. According to the phylogenetic analysis, the TRs of Solanaceae were definitively classified into two groups. One group (Group 1) was the tropine-forming reductases (TRI), in which the functionally identified TRI proteins from *D*. *stramonium* (Portsteffen et al., 1994) and *H*. *niger* (Nakajima et al., 1993a) were included; the other one (Group 2) was the pseudotropine-forming reductases (TRII), including the TRII proteins functionally characterized from *D*. *stramonium* (Portsteffen et al., 1994) and *H*. *niger* (Nakajima et al., 1993a). SlTRI was grouped into the TRI clade according to the phylogenetic analysis we performed (**Figure 4**). Interestingly, the TR from *Cochlearia* *officinalis* (CoTR), which belongs to Cruciferae plant family (Brock et al., 2008), formed an independent cluster (Group 3) that was separated from either the TRI or TRII group. In fact, CoTR was a non-specific TR that catalyzed tropinone, to yield both tropine and pseudotropine (Brock et al., 2008). *SlTRI* was deposited in GenBank with the accession No. MF578231.

### Tissue Patterns of the TA Biosynthesis Genes and Tropane Alkaloids in *S*. *lurida*

**FIGURE 5:**
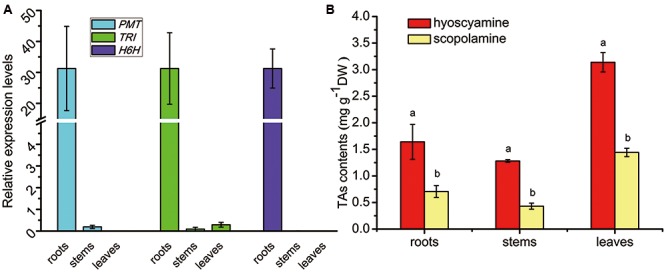
Tissue profiles of the TA biosynthesis genes **(A)** and TA patterns **(B)** in *S. lurida*. The vertical bars represent means ± standard errors (*n* = 3). In **(B)** the different letters indicate a significant difference between means for a given plant organ (as determined by the Duncan test).

The tissue profiles of the three genes involved in TA biosynthesis (*SlPMT*, *SlTRI*, and *SlH6H*) were analyzed in *S*. *lurida* (**Figure 5A**). These genes showed a near-specific expression in the roots. *SlPMT*, the first committed-enzyme, had a very high expression level in the roots that was 164-fold that seen in the stems. *SlPMT* was not expressed in leaves, however. *SlH6H*, the last-committed enzyme, was exclusively expressed in the roots. *SlTRI* was preferentially expressed in roots, and its expression in leaves and stems were barely detected. Since the tissue profile of *SlTRI* was similar to that of *SlPMT* and *SlH6H*, it is not unreasonable to expect SlTRI’s involvement in the tropine-derived alkaloid biosynthesis. The tissue distribution patterns of hyoscyamine and scopolamine were analyzed by HPLC (**Figure 5B**). Both hyoscyamine and scopolamine were detected in the roots, stems, and leaves of *S*. *lurida*, a species that features the hyoscyamine-rich chemotype much like *A*. *belladonna* does (Xia et al., 2016). In each plant organ, hyoscyamine was the main alkaloid occurring with the highest content. In the roots and stems, the hyoscyamine content doubled the scopolamine content; in the leaves, the hyoscyamine content (3.13 mg g^-1^ DW) was more than twice the scopolamine content (1.44 mg g^-1^ DW). Additionally, the hyoscyamine content peaked in the leaves, suggesting that they were the main storage organs for alkaloids in *S*. *lurida*.

### Enzymatic Assays of the Recombinant SlTRI

**FIGURE 6:**
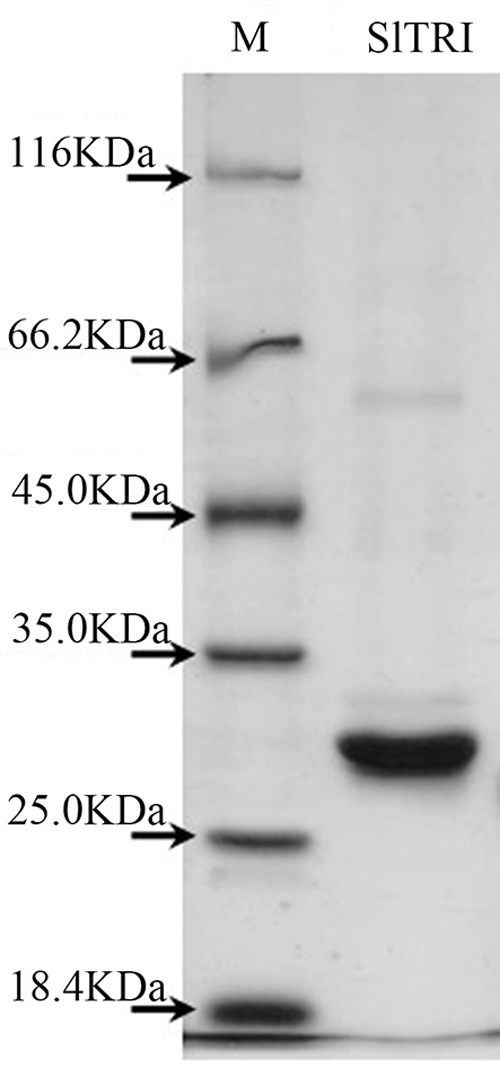
The purified recombinant SlTRI from the engineered *Escherichia* *coli*. M, the standard marker of the proteins; SlTRI, the purified recombinant SlTRI.

**FIGURE 7:**
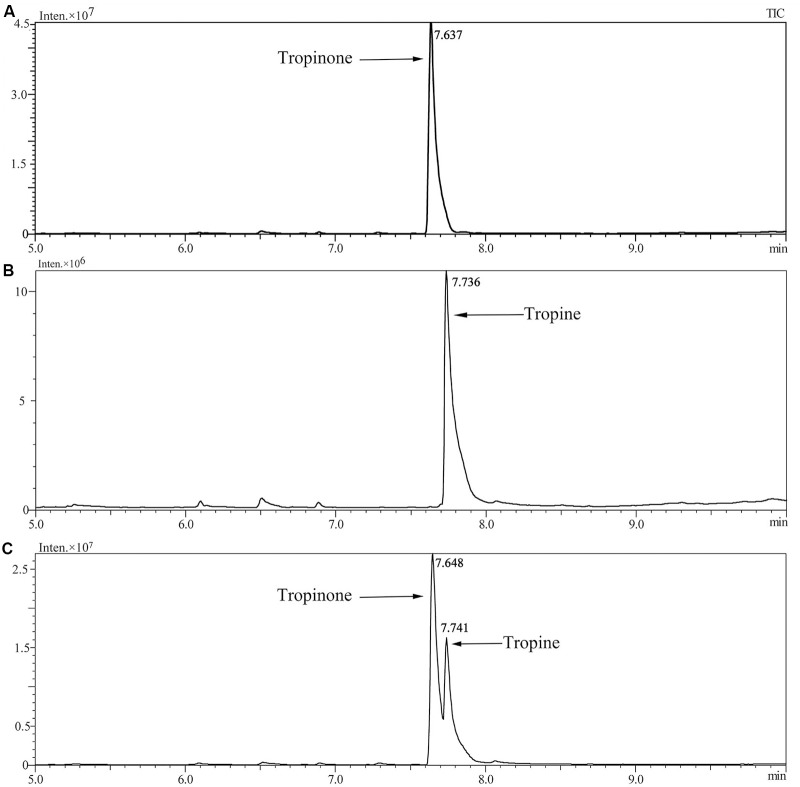
Confirmation of tropine by the GC-MS analysis. **(A)** The GC trace of authentic tropinone; **(B)** the GC trace of authentic tropine; **(C)** the GC trace of tropine as produced by SlTRI in this study.

**FIGURE 8:**
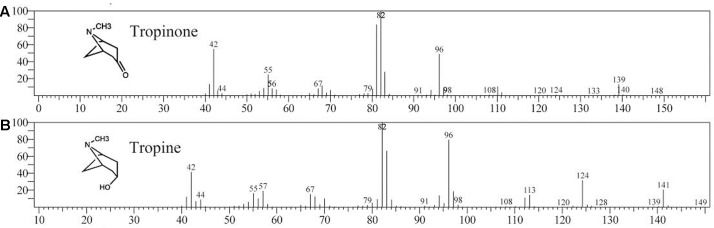
The MASS spectrum of tropinone **(A)** and tropine **(B)**.

Enzymatic assays were performed to identify the functions of SlTRI. The recombinant His-tagged SlTRI was purified (**Figure 6**) from the engineered *E*. *coli*. On the SDS-PAGE gel, the molecular weight of this recombinant SlTRI was c. 29 kDa, consistent with its calculated molecular weight. The purified recombinant protein was used for the enzymatic assay. When tropinone was fed to the recombinant SlTRI, SlTRI catalyzed the reduction of tropinone to form tropine, as confirmed by GC-MS. As **Figure 7** shows, compared to the spectrum of standards (**Figures 7A,B**), the retention time for the tropine produced by SlTRI (7.741 min) was consistent with that of authentic tropine (7.736 min) (**Figure 7C**). The chemical structure was characterized by the MS spectrometric data, together with the Kovats retention indices (RI), quasi-molecular ions ([M]^+^), and characteristic ions with a relative intensity (**Figure 8**). The two compounds, including tropinone and tropine, were, respectively, identified through their comparison with published data (Zayed and Wink, 2004) and the NIST databases. Further, we also analyzed the enzymatic kinetics of the SlTRI. The *K*_m_, *V*_max_, *K*_cat_, and *K*_cat_/*K*_m_ values of SlTRI were, respectively, 40.90 ± 7.059 mM, 32.43 ± 3.175 nkat mg^-1^, 0.98 ± 0.096 s^-1^, and 23.96 s^-1^ M^-1^ at pH 6.4 (**Table 2**). The *K*_m_ value of SlTRI was much higher than that of DsTRI, which includes the purified TRI (1.3 mM) from *D*. *stramonium* (also assayed at pH 6.4) (Portsteffen et al., 1994) and its recombinant TRI (4.18 mM at pH 6.4) (Qiang et al., 2016). The *K*_m_ value of the recombinant SlTRI was 9.78-fold that of the recombinant DsTRI, suggesting the lower affinity of SlTRI for tropinone. By contrast, both the *V*_max_ and *K*_cat_ values of SlTRI were significantly lower than those of DsTRI, thus indicating that SlTRI has the lower catalytic activity than does DsTRI.

**Table 2:** The SlTRI enzymatic kinetics.

Enzymes	Substrate	pH assay	*K*_m_ (mM)	*V*_max_ (nkat⋅mg^-1^)	*K*_cat_ (s^-1^)	*K*_cat_/*K*_m_ (s^-1^⋅M^-1^)
SlTRI	Tropinone	6.4	40.90 ± 7.059	32.43 ± 3.175	0.98 ± 0.096	23.96

### Overexpressing SlTRI Enhanced the TA Biosynthesis

**FIGURE 9:**
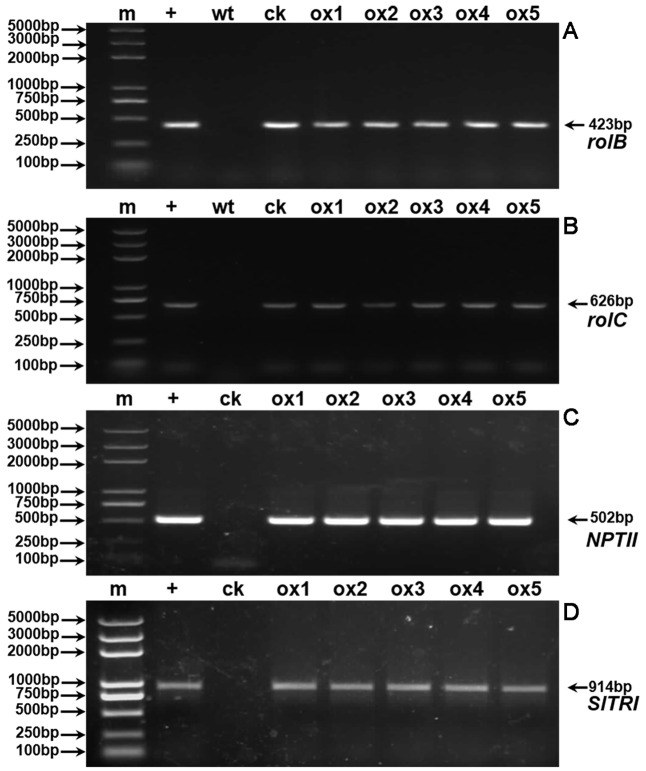
PCR detection of *rolB* **(A)**, *rolC* **(B)**, *NPTII* **(C)**, and *SlTRI* **(D)**. m, DNA marker; +, positive control; wt, wild-type roots of *S. lurida*; ck, hairy roots of *S*. *lurida*; ox, *SlTRI*-overexpressing hairy roots of *S*. *lurida*.

**FIGURE 10:**
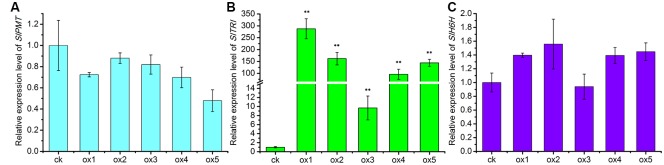
The relative expression levels of the TA biosynthesis genes in the root cultures of *S. lurida*. **(A)** *SlPMT* expression levels; **(B)** *SlTRI* expression levels; **(C)** *SlH6H* expression levels. ^∗∗^Indicates a significant difference from the control at the level of *P* < 0.01. The vertical bars represent means ± standard errors (*n* = 3). ck, the control root culture; ox, the transgenic root cultures with the SlTRI overexpression.

**FIGURE 11:**
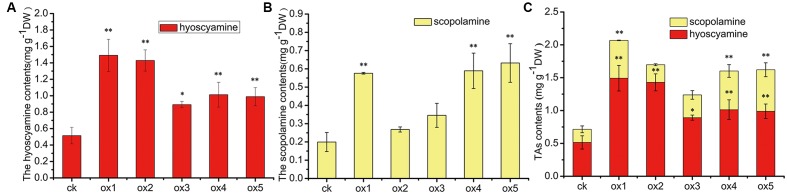
The contents of TAs in the root cultures of *S. lurida*. **(A)** The hyoscyamine contents; **(B)** The scopolamine contents; **(C)** The contents of TAs. ^∗^Indicates a significant difference from the control at the level of *P* < 0.05; ^∗∗^likewise, but at *P* < 0.01. The vertical bars represent means ± standard errors (*n* = 3). ck, the control root culture; ox: the transgenic root cultures with the SlTRI overexpression.

Expression of the rooting genes (i.e., *rolB* and *rolC*) was detected in the hairy roots and the positive control C58C1 (pRiA4), but it was not detected in the wild-type roots of *S*. *lurida*. The fragments of *rolB* and *rolC* were, respectively, 423 bp (**Figure 9A**) and 626 bp (**Figure 9B**). Transgenic hairy root cultures (i.e., overexpressing SlTRI) were confirmed by PCR amplification of the 502-bp *NPTII* gene fragment (**Figure 9C**). The 914-bp PCR products, including the fragments from the 35S promoter and *SlTRI*, were specifically amplified from the positive control and transgenic root cultures (**Figure 9D**). Compared with the control, the expression level of *SlTRI* in all the *SlTRI*-overexpressing root cultures was dramatically higher (9–287-fold greater; **Figure 10B**). At the same time, the expression of *SlPMT* (**Figure 10A**) and *SlH6H* (**Figure 10C**) were also detected in the root cultures. In most cases, each of the two genes (*SlPMT* and *SlH6H*) showed no significant different gene expression between the transgenic root cultures and the control. This suggested that the enzymatic step defined by TRI was substantially improved at the transcriptional level in the transgenic root cultures. The hyoscyamine content in *SlTRI*-overexpressing root lines ranged from 0.892 DW to 1.492 mg g^-1^ DW (**Figure 11A**), with an averaged 1.163 mg g^-1^ DW, whereas the average content of hyoscyamine in the control was 0.515 mg g^-1^ DW (**Figure 11A**). In the *SlTRI*-overexpressing root cultures, the hyoscyamine contents were 1.7- to 2.9-fold that found in the control, with a difference that was significant. On average, the detected scopolamine content was 0.482 mg g^-1^ DW in the *SlTRI*-overexpressing root culture lines (range: 0.268–0.632 mg g^-1^ DW), for which most had significantly higher scopolamine contents than did the control (0.199 mg g^-1^ DW) (**Figure 11B**). Moreover, the total amount of both hyoscyamine and scopolamine was notably higher in the *SlTRI*-overexpressing root lines (range: 1.238–2.068 mg g^-1^ DW) than that in control (0.714 mg g^-1^ DW) (**Figure 11C**). These results suggested that overexpressing SlTRI significantly promoted tropane alkaloid production in the hairy root cultures of *S*. *lurida*.

## Discussion

The biosynthesis of plant secondary metabolites is spatially and temporally regulated. Typically, the tropane alkaloids, including hyoscyamine and scopolamine, are synthesized in the roots of Solanaceae plant species due to the root-specific expression of the TA biosynthesis genes (Flores and Martin-Tanguy, 1991). However, both *H.* *senecionis* (Dehghan et al., 2013) and *Brugmansia arborea* (Qiang et al., 2015) reportedly have an efficient conversion of hyoscyamine to scopolamine in their leaves, because the *H6H* gene is also highly expressed in these organs. The root-specific preferential pattern of the three TA biosynthesis genes (*PMT*, *TRI,* and *H6H*) suggested that the root organ of *S*. *lurida* is where TA biosynthesis occurs. A similar root specific expression pattern was conserved in many TA-producing plant species of Solanaceae, such as *A*. *belladonna* (Xia et al., 2016), *H*. *niger* (Nakajima and Hashimoto, 1999), and *D*. *stramonium* (Patterson and O’Hagan, 2002). Obviously, this kind of coordinated gene expression pattern facilitated the TA biosynthesis in root. The alkaloid analysis showed the leaves of *S*. *lurida* contained much higher levels of both hyoscyamine and scopolamine than did the roots. This result suggests the TAs were synthesized in the roots and moved up to the leaves. Compared with the alkaloid content (1.5% DW) reported by Mills and Jackson (1972), the alkaloid content in our study was about fivefold lower. This could be explained by very young plant material of *S*. *lurida* used in this study, in contrast to the mature plants used by Mills and Jackson. Although very young leaves of *S*. *lurida* were used for the alkaloid analysis, the TA contents were much higher than that (0.278% DW) found in mature leaves of *A*. *belladonna* (Xia et al., 2016). As such, our results confirm that *S*. *lurida* is one of the most effective producers of TAs (Jovanović et al., 1991).

The bioinformatics analysis showed that SlTRI belonged to the family of SDRs and had an amino acid sequence level similar to the SDRs, and that the conserved NADPH-binding site and the catalytic tetrad motif (Oppermann et al., 2003) were present in SlTRI. The phylogenetic analysis definitively revealed the SlTRI as clustered with the functionally identified TRI proteins. Further, the bifunctional CoTR might be an ancient form and thus a possible ancestor of TR. Through gene duplication and mutation, the ancient form of TRs might have evolved into the stereo-specific TRI and TRII (Kushwaha et al., 2013), respectively, producing tropine and pseudotropine in the TA-producing plant species of Solanaceae. Based on the results of enzymatic assay, SlTRI converted tropinone to tropine. Because tropine was the key intermediate of hyoscyamine and scopolamine, we concluded that SlTRI was a tropine-forming reductase participating in the biosynthesis of pharmaceutical tropane alkaloids (including those of hyoscyamine and scopolamine). The *K*_m_ value of SlTRI greatly exceeded that of TRI purified from *D*. *stramonium* (Portsteffen et al., 1994). One possible explanation for this result is that we used recombinant enzymes, whereas the TRI enzyme of *D*. *stramonium* was directly purified from root cultures (Portsteffen et al., 1994). In fact, the *K*_m_ value of the recombinant TRI of *D*. *stramonium* (4.18 mM at pH 6.4) (Qiang et al., 2016) was 3.22-fold higher than that of purified TRI from *D*. *stramonium* root cultures (1.3 mM at pH 6.4). The higher *K*_m_ value of SlTRI, as well as its lower *V*_max_ and *K*_cat_ values, reflects SlTRI’s lower affinity and reductase activity for tropinone relative to DsTRI. From these results, we deduced that SlTRI may be a rate-limiting enzyme in the TA biosynthesis of *S*. *lurida*.

Metabolic engineering is a powerful tool for producing high-value secondary metabolites by genetically modifying pathways *in planta* (Lan et al., 2013). The most frequently used strategy, for devising such an engineered pathway, usually focuses on breaking through the rate-limiting reactions by overexpressing the corresponding enzymes. As the first rate-limiting enzyme, PMT was overexpressed in *H.* *muticus* to enhance its TA biosynthesis (Moyano et al., 2003). Overexpressing H6H, the last rate-limiting enzyme involved in the TA biosynthetic pathway, remarkably improved the scopolamine biosynthesis and production in the TA-producing plant species of Solanaceae (Palazón et al., 2003; Xia et al., 2016). To date, most of the engineering studies of TA biosynthesis are concerned with the *PMT* and *H6H* genes (Ullrich et al., 2017). Tropinone reduction represents the branch point of TA biosynthesis; so, theoretically, genetic modification of the TR-catalyzed steps could significantly affect the TA biosynthesis. To investigate this, we established root cultures with SlTRI overexpression in our present study. In other work, overexpression of TRI increased the contents of hyoscyamine and scopolamine in most of the transgenic hairy root lines of *A*. *belladonna* and *Anisodus acutangulus* when cultured in Gamborg’s B5 liquid medium, and only several transgenic root lines produced the total TAs at higher levels (Richter et al., 2005; Xia et al., 2016). Similar results were obtained in this present study. Without exception, the transgenic hairy root lines of *S*. *lurida* with *SlTRI* overexpression produced hyoscyamine at higher levels (1.7- to 2.9-fold) than did the control root lines. The *SlTRI*-overexpressing root cultures of *S*. *lurida* produced hyoscyamine (1.163 mg/g DW, on average) on par with that produced by (1.376 mg/g DW, on average) the *TRI*-overexpressing root lines of *A. acutangulus* (Kai et al., 2011). In most of our *SlTRI*-overexpressing root lines, the scopolamine contents were also significantly improved, leading to an average content (0.482 mg/g DW) that was almost eightfold that (0.061 mg/g DW) found in the *TRI*-overexpressing root lines of *A. acutangulus* (Kai et al., 2011). When the production of hyoscyamine and scopolamine were pooled, the content of total TAs were much higher in the *SlTRI*-overexpressing root cultures than in the control counterparts. According to a prior report (Richter et al., 2005), as well as this study, we conclude that enhancing the TRI expression through the overexpression method promoted the biosynthesis of TAs.

## Author Contributions

KZ, JZ, and TZ conducted the experiments, including the planting, gene cloning, protein recombination, enzymatic assays, molecular analysis, and alkaloid detection. FQ and XQL participated in the enzymatic assays. CY established the root cultures. MC analyzed the GC-MS data. XZL and ZL conceived the experiments and wrote the manuscript. HZ revised the manuscript. All authors reviewed the manuscript.

## Conflict of Interest Statement

The authors declare that the research was conducted in the absence of any commercial or financial relationships that could be construed as a potential conflict of interest.

## Footnotes

1 http://blast.ncbi.nlm.nih.gov/Blast.cgi

## References

[B1] Brock A. Brandt W. Dräger B. (2008). The functional divergence of short-chain dehydrogenases involved in tropinone reduction. *Plant J.* 54 388–401. 10.1111/j.1365-313X.2008.03422.x 18221363

[B2] Dehghan E. Shahriari Ahmadi F. Ghotbi Ravandi E. Reed D. W. Covello P. S. Bahrami A. R. (2013). An atypical pattern of accumulation of scopolamine and other tropane alkaloids and expression of alkaloid pathway genes in *Hyoscyamus senecionis*. *Plant Physiol. Biochem.* 70 188–194. 10.1016/j.plaphy.2013.05.007 23786817

[B3] Dräger B. (2006). Tropinone reductases, enzymes at the branch point of tropane alkaloid metabolism. *Phytochemistry* 67 327–337. 10.1016/j.phytochem.2005.12.001 16426652

[B4] Dräger B. Hashimoto T. Yamada Y. (1988). Pseudotropine forming tropinone reductase from *Hyoscyamus niger* root cultures. *Agric. Biol. Chem.* 52 2663–2667. 10.1271/bbb1961.52.2663

[B5] Flores H. E. Martin-Tanguy J. (1991). “Polyamines and plant secondary metabolites,” in *Biochemistry and Physiology of Polyamines in Plant*, eds Slocum R. D. Flores H. E. (Boca Raton, FL: CRC Press), 57–96.

[B6] Jakabová S. Vincze L. Farkas Á. Kilár F. Boros B. Felinger A. (2012). Determination of tropane alkaloids atropine and scopolamine by liquid chromatography-mass spectrometry in plant organs of *Datura* species. *J. Chromatogr. A* 1232 295–301. 10.1016/j.chroma.2012.02.036 22391493

[B7] Jovanović V. Grubišić D. Giba Z. Menković N. Ristić M. (1991). Alkaloids in hairy root cultures of *Anisodus luridus*. *Planta Med.* 57 A102. 10.1055/s-2006-960385

[B8] Kai G. Yang S. Luo X. Zhou W. Fu X. Zhang A. (2011). Co-expression of AaPMT and AaTRI effectively enhances the yields of tropane alkaloids in *Anisodus acutangulus* hairy roots. *BMC Biotechnol.* 11:43. 10.1186/1472-6750-11-43 21526999 PMC3111346

[B9] Kushwaha A. K. Sangwan N. S. Trivedi P. K. Negi A. S. Misra L. Sangwan R. S. (2013). Tropine forming tropinone reductase gene from *Withania somnifera* (Ashwagandha): biochemical characteristics of the recombinant enzyme and novel physiological overtones of tissue-wide gene expression patterns. *PLOS ONE* 8:e74777. 10.1371/journal.pone.0074777 24086372 PMC3783447

[B10] Lan X. Chang K. Zeng L. Liu X. Qiu F. Zheng W. (2013). Engineering salidroside biosynthetic pathway in hairy root cultures of *Rhodiola crenulata* based on metabolic characterization of tyrosine decarboxylase. *PLOS ONE* 8:e75459. 10.1371/journal.pone.0075459 24124492 PMC3790822

[B11] Larkin M. A. Blackshields G. Brown N. P. Chenna R. Mcgettigan P. A. McWilliam H. (2007). Clustal W and Clustal X version 2.0. *Bioinformatics* 23 2947–2948. 10.1093/bioinformatics/btm404 17846036

[B12] Mills D. E. Jackson B. P. (1972). *Scopolia lurida*, Dunal; the structure of the leaves and stem. *J. Pharm. Pharmacol.* 24 235–242. 10.1111/j.2042-7158.1972.tb08970.x 4402784

[B13] Moyano E. Jouhikainen K. Tammela P. Palazón J. Cusidó R. M. Piñol M. T. (2003). Effect of pmt gene overexpression on tropane alkaloid production in transformed root cultures of *Datura metel* and *Hyoscyamus muticus*. *J. Exp. Bot.* 54 203–211. 10.1093/jxb/54.381.203 12493848

[B14] Nakajima K. Hashimoto T. (1999). Two tropinone reductases, that catalyze opposite stereospecific reductions in tropane alkaloid biosynthesis, are localized in plant root with different cell-specific patterns. *Plant Cell Physiol.* 40 1099–1107. 10.1093/oxfordjournals.pcp.a029494 10635114

[B15] Nakajima K. Hashimoto T. Yamada Y. (1993a). cDNA encoding tropinone reductase-II from *Hyoscyamus niger*. *Plant Physiol.* 103 1465–1466. 10.1104/pp.103.4.1465 8290643 PMC159147

[B16] Nakajima K. Hashimoto T. Yamada Y. (1993b). Two tropinone reductases with different stereospecificities are short-chain dehydrogenases evolved from a common ancestor. *Proc. Natl. Acad. Sci. U.S.A.* 90 9591–9595. 10.1073/pnas.90.20.9591 8415746 PMC47615

[B17] Nakajima K. Oshita Y. Yamada Y. Hashimoto T. (1999). Insight into the molecular evolution of two tropinone reductases. *Biosci. Biotechnol. Biochem.* 63 1819–1822. 10.1271/bbb.63.1819 10586510

[B18] Oppermann U. Filling C. Hult M. Shafqat N. Wu X. Lindh M. (2003). Short-chain dehydrogenases/reductases (SDR): the 2002 update. *Chem. Biol. Interact.* 143–144, 247–253. 10.1016/S0009-2797(02)00164-3 12604210

[B19] Palazón J. Moyano E. Cusidó R. M. Bonfill M. Oksman-Caldentey K. M. Piñol M. T. (2003). Alkaloid production in *Duboisia* hybrid hairy roots and plants overexpressing the h6h gene. *Plant Sci.* 165 1289–1295. 10.1016/S0168-9452(03)00340-6

[B20] Patterson S. O’Hagan D. (2002). Biosynthetic studies on the tropane alkaloid hyoscyamine in *Datura stramonium*; hyoscyamine is stable to in vivo oxidation and is not derived from littorine via a vicinal interchange process. *Phytochemistry* 61 323–329. 10.1016/S0031-9422(02)00200-5 12359518

[B21] Portsteffen A. Dräger B. Nahrstedt A. (1994). The reduction of tropinone in *Datura stramonium* root cultures by two specific reductases. *Phytochemistry* 37 391–400. 10.1016/0031-9422(94)85066-6 7765621

[B22] Qiang W. Hou Y. L. Li X. Xia K. Liao Z. H. (2015). Cloning and expression of the key enzyme hyoscyamine 6 beta-hydroxylase gene (DaH6H) in scopolamine biosynthesis of *Datura arborea*. *Yao Xue Xue Bao* 50 1346–1355. 26837185

[B23] Qiang W. Xia K. Zhang Q. Zeng J. Huang Y. Yang C. (2016). Functional characterisation of a tropine-forming reductase gene from *Brugmansia arborea*, a woody plant species producing tropane alkaloids. *Phytochemistry* 127 12–22. 10.1016/j.phytochem.2016.03.008 26988730

[B24] Qin B. Ma L. Wang Y. Chen M. Lan X. Wu N. (2014). Effects of acetylsalicylic acid and UV-B on gene expression and tropane alkaloid biosynthesis in hairy root cultures of *Anisodus luridus*. *Plant Cell Tissue Organ Cult.* 117 483–490. 10.1007/s11240-014-0454-z

[B25] Richter U. Rothe G. Fabian A. K. Rahfeld B. Dräger B. (2005). Overexpression of tropinone reductases alters alkaloid composition in *Atropa belladonna* root cultures. *J. Exp. Bot.* 56 645–652. 10.1093/jxb/eri067 15642710

[B26] Tamura K. Peterson D. Peterson N. Stecher G. Nei M. Kumar S. (2011). MEGA5: molecular evolutionary genetics analysis using maximum likelihood, evolutionary distance, and maximum parsimony methods. *Mol. Biol. Evol.* 28 2731–2739. 10.1093/molbev/msr121 21546353 PMC3203626

[B27] Ullrich S. F. Hagels H. Kayser O. (2017). Scopolamine: a journey from the field to clinics. *Phytochem. Rev.* 16 333–353. 10.1007/s11101-016-9477-x

[B28] Wang X. Chen M. Yang C. Liu X. Zhang L. Lan X. (2011). Enhancing the scopolamine production in transgenic plants of *Atropa belladonna* by overexpressing pmt and h6h genes. *Physiol. Plant.* 143 309–315. 10.1111/j.1399-3054.2011.01506.x 21883248

[B29] Wang Y. Meng L. L. Yang Y. P. Duan Y. W. (2010). Change in floral orientation in *Anisodus luridus* (Solanaceae) protects pollen grains and facilitates development of fertilized ovules. *Am. J. Bot.* 97 1618–1624. 10.3732/ajb.1000010 21616797

[B30] Xia K. Liu X. Zhang Q. Qiang W. Guo J. Lan X. (2016). Promoting scopolamine biosynthesis in transgenic *Atropa belladonna* plants with pmt and h6h overexpression under field conditions. *Plant Physiol. Biochem.* 106 46–53. 10.1016/j.plaphy.2016.04.034 27135818

[B31] Yun D.-J. Hashimoto T. Yamada Y. (1992). Metabolic engineering of medicinal plants: transgenic *Atropa belladonna* with an improved alkaloid composition (scoolamlne/hyoscyamine 61-hydroxylase). *Appl. Biol. Sci.* 89 11799–11803. 10.1073/pnas.89.24.11799 1465402 PMC50644

[B32] Zayed R. Wink M. (2004). Induction of tropane alkaloid formation in transformed root cultures of *Brugmansia suaveolens* (Solanaceae). *Z. Naturforsch. C* 59 863–867. 10.1515/znc-2004-11-1216 15666547

[B33] Zeng J. Zhao K. Lan X. Liao Z. (2016). Analyzing the contents of tropane alkaloids in *Scopolia lurida*, a resource plant species of Tibetan medicines. *Sci. Technol. Tibet.* 279 60–62. 10.3732/ajb.1000010 21616797

[B34] Zhang L. Kai G.-Y. Lu B.-B. Zhang H.-M. Tang K.-X. Jiang J.-H. (2005). Metabolic engineering of tropane alkaloid biosynthesis in plants. *J. Integr. Plant Biol.* 47 136–143. 10.1111/j.1744-7909.2005.00024.x

[B35] Zhang L. Yang B. Lu B. Kai G. Wang Z. Xia Y. (2007). Tropane alkaloids production in transgenic *Hyoscyamus niger* hairy root cultures over-expressing Putrescine N-methyltransferase is methyl jasmonate-dependent. *Planta* 225 887–896. 10.1007/s00425-006-0402-1 17004056

